# Hospital Readmissions After Vertebral Fracture in Older Adults: A Comparison of Management Strategies

**DOI:** 10.7759/cureus.103704

**Published:** 2026-02-16

**Authors:** Nathan Gilmore, Sebastian Leal, Antonio Da Costa, Joseph Ouslander, Gabriella Engstrom

**Affiliations:** 1 Medicine, Florida Atlantic University Charles E. Schmidt College of Medicine, Boca Raton, USA; 2 Medicine, Florida International University, Herbert Wertheim College of Medicine, Miami, USA; 3 Geriatric Medicine, Florida Atlantic University Charles E. Schmidt College of Medicine, Boca Raton, USA

**Keywords:** balloon kyphoplasty, conservative management, hospital readmission, vertebral fractures, vertebroplasty

## Abstract

Background: Osteoporotic vertebral fractures are common in older adults and are associated with pain, impaired mobility, and increased healthcare utilization. Balloon kyphoplasty and vertebroplasty are minimally invasive treatment options, but their association with short-term hospital utilization is less clear. This study evaluated whether treatment modality, age, and body mass index are associated with 30-day hospital return among adults aged 75 years and older hospitalized with vertebral fractures.

Methods: This retrospective secondary analysis used a quality improvement database from a community-based teaching hospital and included admissions from 2017 to 2023 for patients aged 75 years and older with a primary diagnosis of vertebral fracture. Patients who died during hospitalization or were discharged to hospice were excluded. Patients were grouped by treatment: balloon kyphoplasty, vertebroplasty, or conservative management. The primary outcome was hospital return within 30 days, defined as a post-discharge event resulting in readmission, emergency department visit, or observation stay. Descriptive statistics, chi-square and t-tests, and logistic regression were used to evaluate associations between treatment type, body mass index, age, and 30-day hospital return, with statistical significance set at p < 0.05.

Results: There was no statistically significant difference in 30-day hospital return between patients treated with balloon kyphoplasty or vertebroplasty and those managed conservatively. Body mass index and age were not significant predictors of 30-day hospital return in this cohort. A subset of admissions had missing body mass index values, including two patients in the balloon kyphoplasty or vertebroplasty group who did not return, and 58 patients in the conservative management group who did not return.

Conclusions: Among adults aged 75 years and older hospitalized with vertebral fracture, balloon kyphoplasty and vertebroplasty were not associated with lower 30-day hospital return compared with conservative management. In this dataset, neither body mass index nor age meaningfully predicted short-term hospital return, suggesting that factors beyond these patient characteristics and initial treatment modality may drive early utilization after discharge.

## Introduction

Osteoporotic vertebral fractures are among the most common fragility fractures in older adults and are associated with pain, functional decline, impaired mobility, and increased healthcare utilization. Lifetime risk estimates suggest that a substantial proportion of adults over the age of 50 years will experience an osteoporotic fracture, with vertebral fractures contributing significantly to the overall disease burden. Population-based studies demonstrate that vertebral fracture prevalence increases sharply with advancing age in both men and women, reflecting the intersection of skeletal fragility, multimorbidity, and physiologic vulnerability in later life [1-3]. In the United States, osteoporosis-related fractures account for tens of billions of dollars in annual healthcare costs, a figure projected to rise substantially as the population continues to age [4,5].

Management strategies for vertebral fractures include conservative management, such as analgesia, bracing, and early mobilization, as well as minimally invasive vertebral augmentation procedures, including balloon kyphoplasty and vertebroplasty. Balloon kyphoplasty involves percutaneous insertion of an inflatable balloon tamp to restore vertebral body height prior to cement injection, whereas vertebroplasty consists of direct percutaneous injection of polymethylmethacrylate cement without cavity creation [6-10]. Conservative management remains the initial treatment approach for many patients, but may provide inadequate pain relief or functional recovery in cases of severe or persistent symptoms. Vertebral augmentation techniques were developed to address these limitations by stabilizing the fractured vertebral body, reducing pain, and improving mobility [6,7].

The clinical evidence supporting balloon kyphoplasty and vertebroplasty has been mixed. Randomized controlled trials evaluating vertebroplasty demonstrated no significant benefit over sham procedures with respect to pain reduction or functional outcomes [6,7]. These trials prompted significant public and policy response, influencing reimbursement decisions and clinical practice for over a decade, while subsequent critiques highlighted limitations in study design, patient selection, and external validity [6,7]. In contrast, the FREE trial and subsequent studies reported that balloon kyphoplasty was associated with earlier improvements in pain, mobility, and quality of life when compared with nonsurgical management [8,9]. Longer-term comparative analyses suggest that balloon kyphoplasty and vertebroplasty yield broadly similar outcomes with respect to pain relief and disability, with both procedures demonstrating acceptable safety profiles [10]. Observational studies have further suggested a potential association between vertebral augmentation and reduced mortality compared with nonoperative management, although these findings may be influenced by selection bias and unmeasured confounding factors [11].

Despite extensive investigation of pain and functional outcomes, less is known about the relationship between vertebral fracture treatment modality and short-term hospital utilization, particularly 30-day hospital return rates. This outcome is clinically important, as early hospital returns are associated with increased morbidity, disrupted recovery, and higher health care costs in older adults. Prior studies in spine surgery and orthopedic populations have suggested that advanced age and extremes of body mass index may be associated with increased complication rates and hospital readmissions [12-15]. However, the extent to which these factors influence short-term outcomes following vertebral fracture management remains unclear, particularly among older adults treated with balloon kyphoplasty, vertebroplasty, or conservative management.

Given the aging population, the rising prevalence of osteoporotic vertebral fractures, and the growing emphasis on reducing hospital readmissions, evaluating the association between treatment modality, body mass index, and age with 30-day hospital return rates is clinically relevant. Improved understanding of these relationships may help inform treatment selection, guide post-discharge care planning, and identify patients at increased risk for early hospital utilization.

## Materials and methods

This study was a retrospective secondary analysis of data obtained from a quality improvement database at a community-based teaching hospital. The dataset included hospital admissions between 2017 and 2023 and focused on patients aged 75 years and older who were hospitalized with a primary diagnosis of vertebral fracture. The objective of the study was to evaluate the association between treatment modality and 30-day hospital return rates, with treatment categorized as balloon kyphoplasty, vertebroplasty, or conservative management.

Eligible participants included patients aged 75 years or older who were admitted with a primary diagnosis of vertebral fracture. Patients were excluded if they died during the index hospitalization or were discharged to hospice care, as these individuals did not have the potential for post-discharge hospital return and therefore could not contribute to the primary outcome analysis.

Demographic and clinical variables collected included age, body mass index, and treatment type. Patients were assigned to one of three groups based on the treatment received during hospitalization: balloon kyphoplasty, vertebroplasty, or conservative management. Hospital return within 30 days was defined as any post-discharge encounter resulting in hospital readmission, emergency department visit, or observation stay. Outcomes were identified using hospital administrative records and International Classification of Diseases, Tenth Revision codes.

The primary outcome was 30-day hospital return. Secondary variables included patient demographics and treatment modality. Associations between age, body mass index, treatment type, and hospital return were evaluated to identify potential predictors of short-term hospital utilization.

Descriptive statistics were used to summarize patient characteristics across treatment groups. Categorical variables were compared using chi-square tests, and continuous variables were compared using t-tests. Logistic regression analysis was performed to assess the association between treatment modality, body mass index, age, and the likelihood of 30-day hospital return. Statistical significance was defined as a p-value less than 0.05. All analyses were conducted using IBM SPSS Statistics version 29.0 (IBM Corporation, Armonk, NY, USA).

## Results

A total of admissions for patients aged 75 years and older hospitalized with vertebral fractures were included in the analysis. Patients were stratified by treatment modality into conservative management and balloon kyphoplasty or vertebroplasty groups. Baseline demographic and clinical characteristics are summarized in Table 1.

**Table 1:** Characteristics of admissions by treatment modality Baseline demographic and clinical characteristics of hospital admissions for vertebral fracture stratified by conservative management versus balloon kyphoplasty or vertebroplasty. Values are reported as number (percentage) unless otherwise indicated. Laboratory values are reported as mean ± standard deviation. A small proportion of admissions had missing laboratory values.

Characteristics	Conservative Management n (%)	Balloon Kyphoplasty or Vertebroplasty n (%)	P-value
Female sex	274 (74.7%)	21 (84.0%)	0.295
Caucasian race	351 (95.6%)	25 (100.0%)	0.286
Congestive heart failure	44 (12.0%)	5 (20.0%)	0.241
Coronary atherosclerosis	35 (9.5%)	5 (20.0%)	0.094
Atrial fibrillation	73 (19.9%)	6 (24.9%)	0.62
Cerebrovascular disease	6 (1.6%)	0 (0.0)	0.519
Chronic kidney disease	12 (3.3%)	2 (8.0%)	0.218
Diabetes mellitus	28 (7.6%)	2 (8.0%)	0.946
Hypertension	150 (40.9%)	8 (32.0%)	0.382
Osteoporosis	17 (4.6%)	1 (4.0%)	0.884
Rheumatic disease	10 (2.7%)	4 (16.0%)	<0.001
Stroke or transient ischemic attack	8 (2.2%)	0 (0.0)	0.456
Sodium mmol per L mean ± SD	136.7 ± 4.8	136.3 ± 6.0	0.682
Hemoglobin g per dL mean ± SD	12.0 ± 1.9	11.4 ± 1.6	0.14
Potassium mmol per L mean ± SD	4.0 ± 0.5	4.1 ± 0.5	0.578

There were no statistically significant differences between treatment groups with respect to sex, race, or most comorbid conditions. Patients undergoing balloon kyphoplasty or vertebroplasty had a higher prevalence of rheumatic disease compared with those managed conservatively. Laboratory values, including sodium, hemoglobin, and potassium levels, were similar between groups. A small proportion of admissions had missing laboratory data.

Among patients who underwent balloon kyphoplasty or vertebroplasty, there was no significant difference in age or body mass index between those who returned to the hospital within 30 days and those who did not return. Mean age and mean body mass index were comparable between groups, and neither variable was significantly associated with 30-day hospital return in this cohort (Table 2). Two patients in the balloon kyphoplasty or vertebroplasty group who did not return to the hospital had missing body mass index data.

**Table 2:** Comparison of age and body mass index among patients undergoing balloon kyphoplasty or vertebroplasty by 30-day hospital return Comparison of age and body mass index between patients who returned to the hospital within 30 days and those who did not, following balloon kyphoplasty or vertebroplasty. Values are reported as mean ± standard deviation. Two patients who underwent balloon kyphoplasty or vertebroplasty and did not return to the hospital had missing body mass index data.

Variable	Returned to Hospital Within 30 Days	Did Not Return to Hospital Within 30 Days	P-value
Age (years), mean ± SD	84.8 ± 3.4	84.9 ± 4.3	0.962
Body mass index (kg/m^2^), mean ± SD	25.8 ± 5.4	24.7 ± 3.4	0.583

Similarly, among patients managed conservatively, there were no statistically significant differences in age or body mass index between patients who returned to the hospital within 30 days and those who did not. Mean age and mean body mass index were nearly identical between groups, and neither variable was associated with 30-day hospital return (Table 3).

**Table 3:** Comparison of age and body mass index among patients undergoing conservative management by 30-day hospital return Comparison of age and body mass index between patients who returned to the hospital within 30 days and those who did not following conservative management. Values are reported as mean ± standard deviation.

Variable	Returned to Hospital Within 30 Days	Did Not Return to Hospital Within 30 Days	P-value
Age (years), mean ± SD	85.9 ± 6.8	85.9 ± 6.1	0.926
Body mass index (kg/m^2^), mean ± SD	24.7 ± 4.6	24.7 ± 5.2	0.951

In multivariable logistic regression analysis, treatment modality, age, and body mass index were not significant predictors of 30-day hospital return. Balloon kyphoplasty or vertebroplasty was not associated with a lower likelihood of hospital return compared with conservative management.

## Discussion

Vertebral fractures in older adults represent a significant clinical challenge due to their association with pain, functional impairment, and increased healthcare utilization [4,16]. Minimally invasive vertebral augmentation procedures such as balloon kyphoplasty and vertebroplasty were developed to provide structural stabilization of the fractured vertebra and potentially improve pain and mobility more rapidly than conservative measures alone [8,16]. Randomized controlled trials of vertebroplasty have not demonstrated consistent benefit over sham procedures in pain or functional outcomes, raising questions about its routine use [6,7]. Conversely, kyphoplasty has been associated with early improvements in pain and quality of life in some studies, although long-term outcomes appear similar to nonoperative management [8,9]. Observational data have also suggested potential mortality benefits with vertebral augmentation compared with conservative care, though these findings may be susceptible to confounding and selection bias due to the nonrandomized nature of many cohort studies [10,11].

The findings of the current study extend this body of literature by examining the association between treatment modality and short-term healthcare utilization, a clinically relevant outcome that reflects both patient recovery and healthcare system burden [13]. In this cohort of adults aged 75 years and older hospitalized with vertebral fracture, neither balloon kyphoplasty nor vertebroplasty was associated with a statistically significant reduction in 30-day hospital return compared with conservative management. This result aligns with prior observations that procedural interventions may not uniformly translate into measurable improvements in all domains of patient outcomes, particularly when evaluated outside of randomized settings [11,16].

Advanced age and body mass index have been implicated as risk factors for postoperative complications and hospital readmissions following various spinal and orthopedic procedures [12-15]. However, in this study cohort, neither age nor body mass index was significantly associated with 30-day hospital return in either the procedural or conservative management groups. This suggests that these patient characteristics may not be primary drivers of early hospital utilization after vertebral fracture management, at least when considered in isolation [16]. It remains possible that other clinical factors, such as baseline functional status, severity of fracture displacement, vertebral level involvement, pain severity, and comorbid conditions, contribute more substantially to early post-discharge healthcare needs, and these warrant further investigation [3,16].

The absence of a clear association between treatment modality and short-term hospital return does not necessarily imply equivalence of clinical benefit across interventions [11]. Rather, it highlights the complexity of vertebral fracture care in older adults and emphasizes the need for individualized clinical decision-making that weighs the potential benefits of intervention against patient preferences, procedural risk, and resource utilization considerations [3,11]. In particular, selection bias may influence the real-world application of vertebral augmentation, as clinicians and patients may preferentially choose procedural options for those perceived to have greater potential for functional recovery or lower procedural risk [1,10].

This study is subject to limitations inherent to retrospective analyses, including potential unmeasured confounding and reliance on administrative coding for case identification. Detailed clinical data, such as fracture morphology, pain scores, functional outcomes, and precise indications for intervention, were not available, which may limit the ability to discern nuanced treatment effects. Additionally, the cohort reflects admissions at a single community-based teaching hospital, which may constrain generalizability to other practice settings or population subgroups.

Future research should aim to incorporate prospective designs with standardized outcome measures to better understand how vertebral augmentation and conservative strategies impact both short- and long-term patient-centered outcomes [6,7]. Identification of subpopulations that derive the greatest benefit from specific management approaches could improve clinical guidelines and tailored care pathways in this vulnerable population [11,15].

## Conclusions

In this retrospective analysis of adults aged 75 years and older hospitalized with vertebral fracture, treatment with balloon kyphoplasty or vertebroplasty was not associated with a reduction in 30-day hospital return rates compared with conservative management. Additionally, age and body mass index were not significant predictors of early hospital utilization in this cohort. These findings suggest that short-term hospital return after vertebral fracture may be influenced by factors beyond treatment modality and basic demographic characteristics. Future studies incorporating fracture severity, functional status, pain burden, and post-discharge support may help identify patients at increased risk for early hospital return and inform more individualized management strategies.
