# Correction: Hospital Readmissions After Vertebral Fracture in Older Adults: A Comparison of Management Strategies

**DOI:** 10.7759/cureus.c403

**Published:** 2026-02-17

**Authors:** Nathan Gilmore, Sebastian Leal, Antonio Da Costa, Joseph Ouslander, Gabriella Engstrom

**Affiliations:** 1 Medicine, Florida Atlantic University Charles E. Schmidt College of Medicine, Boca Raton, USA; 2 Medicine, Florida International University, Herbert Wertheim College of Medicine, Miami, USA; 3 Geriatric Medicine, Florida Atlantic University Charles E. Schmidt College of Medicine, Boca Raton, USA

Due to a technical error, Antonio Da Costa was accidentally omitted from the final author list. He has now been added as the third author.

